# Scale-Up of Nanocorundum Synthesis by Mechanochemical Dehydration of Boehmite

**DOI:** 10.1021/acs.iecr.4c03537

**Published:** 2025-01-13

**Authors:** Sarah Triller, Amol Amrute, Ferdi Schüth

**Affiliations:** Max-Planck-Institut für Kohlenforschung, Kaiser-Wilhelm-Platz 1, D-45470 Mülheim an der Ruhr, Germany

## Abstract

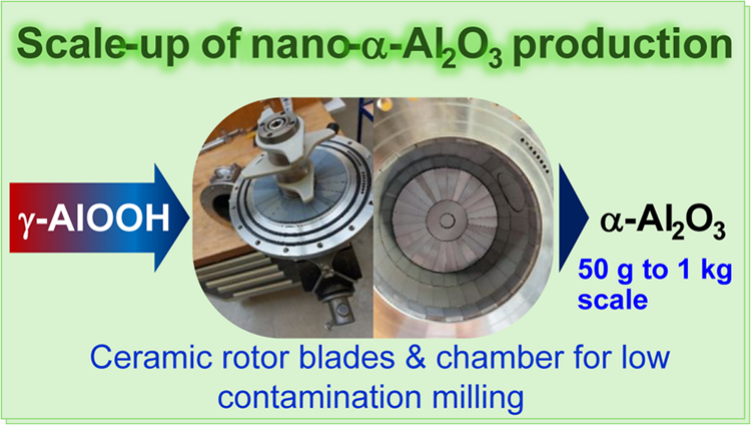

This work presents the scale-up of room-temperature mechanochemical synthesis of nanocorundum (high-surface-area α-Al_2_O_3_) from boehmite (γ-AlOOH). This transformation on the 1 g scale using a laboratory shaker mill had previously been reported. High-energy Simoloyer ball mills equipped with milling chambers of sizes ranging from 1 to 20 L were used to scale up the mechanochemical nanocorundum synthesis to the 50 g to 1 kg scale, which paves the way to further increase batch size. Milling chambers made of steel and lined with silicon nitride (Si_3_N_4_) and milling balls made of steel, zirconia (ZrO_2_), and silicon nitride (Si_3_N_4_) were investigated to address the abrasion problem, leading to contamination of the alumina. Furthermore, several other process parameters, such as ball-to-powder ratio, degree of chamber filling, and milling speed, were optimized to find the conditions for efficient formation of nanocorundum with minimum contamination. Impact forces were found to be decisive in driving the transformation from boehmite to corundum. The nanocorundum produced in the scaled-up experiments has a high specific surface area >110 m^2^/g with an average particle size of ∼13 nm at a low level of contamination. The optimal sample was also shown to possess improved stability of surface area when exposed to temperatures up to 1200 °C. These results successfully demonstrate the scale-up of 1 g scale results to up to the 1 kg scale and may serve as a blueprint for scaling up also other mechanochemistry processes.

## 1 Introduction

Mechanochemistry has recently emerged as a highly effective tool for performing chemical reactions and materials synthesis.^1−7^ It allows reactions under milder conditions and in a solvent-free manner or with very small amounts of solvent, making it often a more sustainable method.^8^ As of today, mechanochemistry has been reported for the solvent-less (solid-state) synthesis of important organic compounds (e.g., via solid-state C–C bond forming reactions, organocatalytic transformations),^9−11^ organometallic or hybrid materials (e.g., MOFs, POMs, surface-functionalized solids),^12−15^ solid inorganic materials (e.g., H_2_ storage materials, single metal oxides, mixed metal oxides, supported (bi)metallic nanoparticles), biomass conversion (e.g., depolymerization of cellulose),^16−18^ and for performing catalytic reactions under milling.^19,20^ Notably, most of these studies have shown promise only on a laboratory scale. However, scale-up of these findings obtained for milligram amounts is scarcely reported. One reason for this might be linked to the fact that these studies are predominantly performed using benchtop equipment, such as planetary ball mills or shaker mills, for which no large-scale equivalents are available. Moreover, there are no straightforward scaling relations, unlike for other processes, which would allow easy transfer of laboratory data to the synthesis of commercially relevant amounts.^21^ Still, some recent studies have shown successful scale-ups of laboratory results, such as the synthesis of MOFs using twin screw and single screw extruders in a continuous mode,^22^ attrition mill depolymerization of lignocellulosic biomass at several hundred grams scale using attrition mill^23^ and on the kilogram scale in a 20 L Simoloyer device,^18^ and synthesis of a TiFeMn alloy for hydrogen storage in a 100 L Simoloyer.^24^

In our recent work, we reported the synthesis of high-surface-area corundum (α-Al_2_O_3_) by mechanochemical dehydration of boehmite (γ-AlOOH).^3^ Corundum in nanocrystalline form is an interesting material for ceramics and catalysis applications, and easy access to it may open up new applications of this alumina polymorph.^5,6,25−27^ However, the scale-up of the synthesis of this hard material in a ball mill is not straightforward. In the reported gram-scale synthesis,^3^ we observed that the boehmite-to-corundum conversion is an impact-driven process, and due to the required high-impact milling and abrasive nature of the formed alumina phase, there is significant contamination of the product alumina by the construction material of milling vessel and balls. For instance, contaminations of 7.3 wt % tungsten and 0.7 wt % cobalt (cobalt is the matrix for the WC) were observed when ball milling was performed for 3 h in a shaker mill using a 25 mL tungsten carbide (WC) vessel and three 12 mm diameter WC balls.^3^ For these experiments, tungsten and cobalt were successfully leached out to relatively low levels by oxidative treatment so that the alumina could be characterized in detail. However, such leaching is not economic when targeting a large-scale synthesis; moreover, low levels of metal contamination can also be detrimental for many applications. Ball milling of boehmite to form corundum in shaker mills using stainless steel vessels and balls also resulted in contamination (iron up to 8 wt %). Planetary mills, in which the formation of corundum from boehmite also takes place, also suffer from abrasion, causing contamination of the target materials from vessels and balls. Besides, as mentioned above, scale-up in shaker or planetary mills is not possible due to their unavailability for milling of larger amounts of material.

It is, therefore, necessary to opt for other types of ball milling devices that are developed for large-scale operation. However, the device also needs to allow high-energy ball milling, since the boehmite to corundum conversion needs a high mechanical energy impact. In addition, the parts of the device in contact with the powder should be available in different construction materials to address the contamination issue. While complete elimination of contamination is virtually impossible in high-energy milling, the availability of different construction materials allows the selection of materials for which contamination is innocent for the target application. Rotary or drum, vibration, attritor, and Simoloyer ball mills are the main types of milling devices available for large amounts of product.^4^ Among them, rotary ball mills are characterized by low-energy input and are typically used for mixing and comminution applications. As a first attempt, we also performed initial trials in a rotary mill and, expectedly, did not observe any conversion of boehmite due to the low-energy input. Likewise, a vibration ball mill with several liters chamber size was also tried. However, it was found that the impact from the collisions of milling balls was not effective for the conversion of boehmite to corundum. Attritors, in general, and Simoloyer are regarded as high-energy devices, and we opted for the latter, thanks to its advanced principle leading to the high-kinetic-energy milling and control over process parameters (see details in Section 4). Moreover, Simoloyer ball mills are available in sizes from 1 to 900 L vessel volumes, allowing a stepwise scale-up; and with different material of vessel and milling media (e.g., various types of steel and ceramics), enabling to deal with transition metal contaminations. The latter is particularly detrimental in catalysis applications due to the often undesired catalytic activity, which may interfere with the performance in the target reaction. Additionally, contamination by steel components may promote sintering in high-temperature uses.

**Figure 1:**
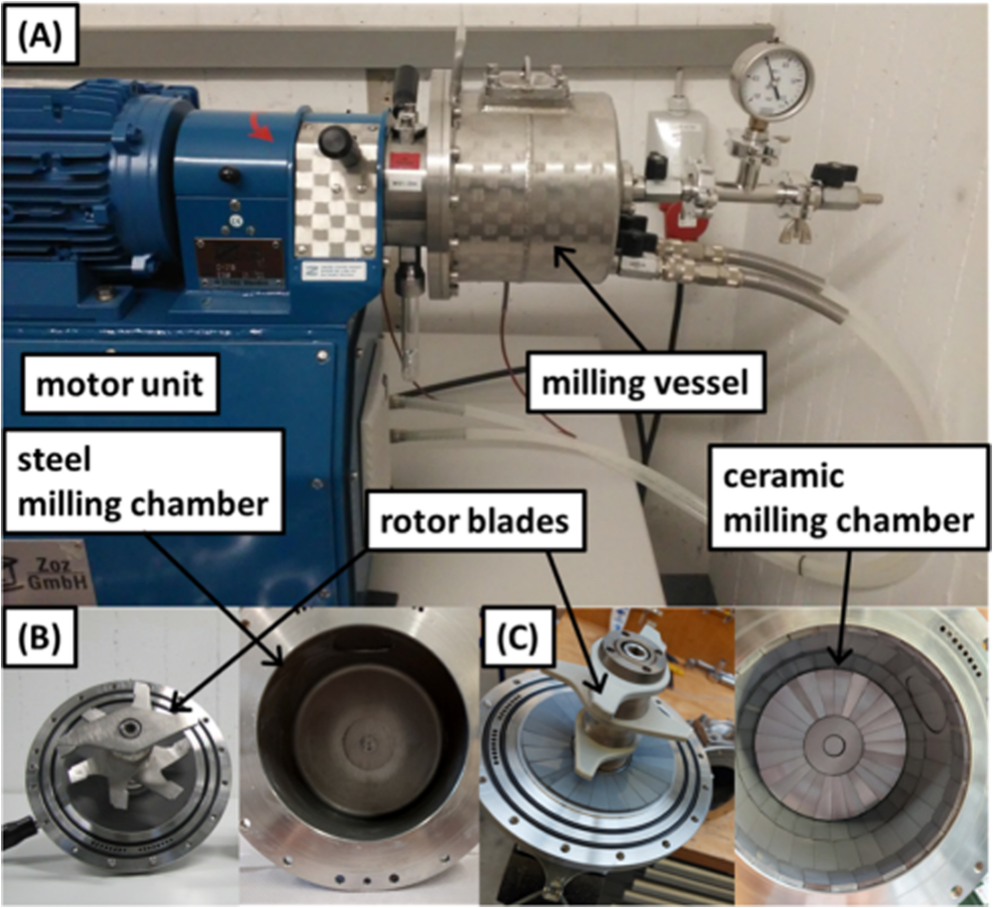
Simoloyer CM01 and milling vessels utilized in this study on CM01. (A) Driving motor of the CM01 unit and a 2 L steel vessel attached. (B) 1 L steel unit from inside. (C) Rotor and vessel of the modified Si_3_N_4_ vessel.

This work reports the scale-up of the synthesis of high-surface-area (HSA) α-Al_2_O_3_ by mechanochemical dehydration of boehmite at room temperature from 1 g to the kg scale in Simoloyer ball mills (Figure 1). The results suggest that high-surface-area corundum production can be successfully scaled up. Moreover, the important aspect of contamination due to abrasion is addressed, a problem that is usually not even mentioned in many research papers. The choice of appropriate materials of milling vessel and balls, and also the larger scale, on the one hand, can reduce contamination; on the other hand, contamination can be directed to less problematic compounds.

## 2 Results and Discussion

### 2.1 Scale-Up of Mechanochemical Boehmite to α-Alumina Synthesis

The scale-up of boehmite to nanocorundum by mechanochemistry was investigated using Simoloyer ball mills with sizes of milling vessels ranging from 1 to 20 L. The experiments involving 1 and 2 L milling vessels were conducted on a machine of the type CM01 (Figure 1), while for 8 and 20 L vessels, we used CM08 and CM20 mills at Zoz GmbH’s process laboratory.

When the batch sizes were changed, the reaction parameters were kept as close to each other as possible. In particular, the rotor tip velocity, filled volume fraction, and ball-to-powder ratio were constant during the conducted scaling-up experiments. The kinetic energy of the balls scales with the rotor tip velocity, which in turn scales with the diameter of the rotor blade, as shown in eq 1.^28^
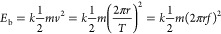
1In this equation, *k* is a prefactor which takes into account the efficiency of energy transfer between rotor and balls, *m* refers to the mass of the balls, *v* is the velocity of the rotor tip, *r* defines the radius of the rotor, *T* gives the time per rotation, with 1/*T* equal to the rotation speed *f*, and E_b_ is the resulting energy of the balls. In a given system with similar parameters as those in our study, the energy of the balls scales with the square of the rotor blade diameter. The rotor tip velocity was kept constant, and therefore, the set milling speeds were 1540, 950, and 625 rpm for the 1, 8, and 20 L systems, respectively.

First, the degree of filling and ball-to-powder ratio was optimized for a small-scale system (see Figures S1 and S2). The progress of the mechanochemical reaction was monitored by powder X-ray diffraction (XRD) of samples taken after suitable times (3, 5, and 8 h). The use of different amounts of boehmite powders (50, 75, and 100 g) does not significantly affect the filling ratio, since the amount of powder is small compared to the amount of balls. For all three amounts of boehmite, full conversion was observed after 8 h of milling. The ball-to-powder (BPR or B:P) mass ratio was in the range of 17.5–25, which was adjusted by the amount of powder and, thus, does not substantially affect the degree of filling and the conversion. However, significant differences were noticed when the degree of filling and the B/P ratio were adjusted by different amounts of milling balls (see Figures S1 and S2 and related explanation). Therefore, in the following the degree of filling and the B:P ratio were kept at the optimized conditions.

**Figure 2:**
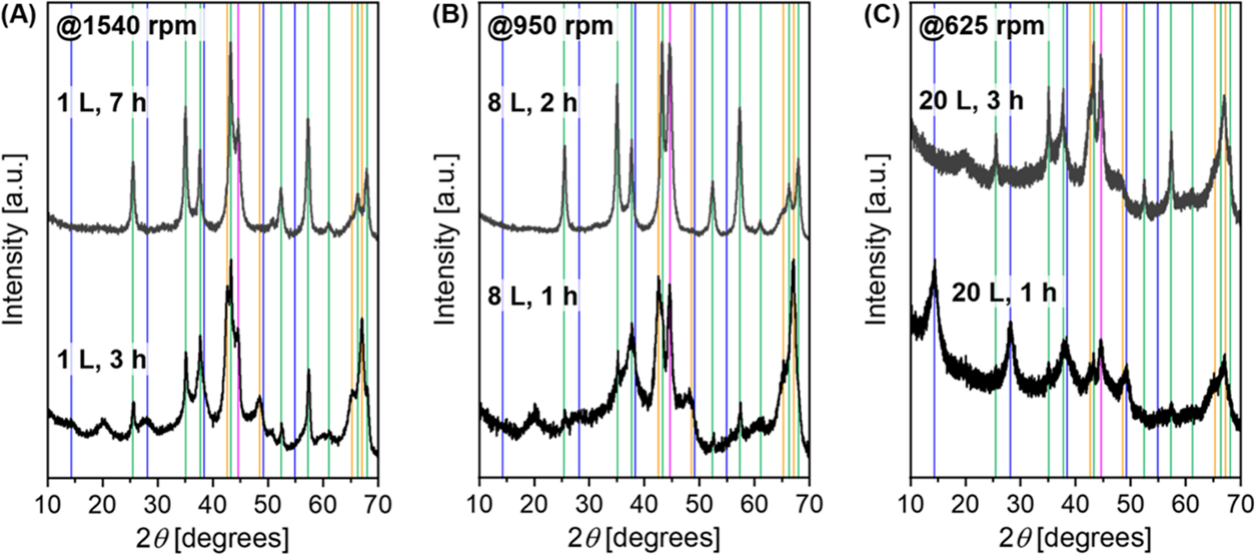
Powder XRD patterns of boehmite samples after ball milling in 1, 8, and 20 L steel vessels using steel balls in a constant ball-to-powder mass ratio (bpr) milled at 1540, 950, and 625 rpm, respectively. The boehmite amount was raised from 50 to 400 and 1000 g to keep the degree of filling of the milling vessels constant. Milling speeds were chosen according to identical rotor tip velocities. All patterns from the samples milled for a longer time show reflections that can be fitted to α-alumina; some additional reflections that belong to the impurity from steel components due to abrasion are also marked. Identified crystalline phases are indicated with vertical lines with color code: blue: boehmite, green: α-alumina, orange: tohdite, pink: iron, and cyan: ZrO_2_.

The XRD patterns of boehmite samples ball milled in milling vessels of different sizes and milling speeds (which are adjusted to keep rotor tip velocity constant for different sizes) are shown in Figure 2. Indicated by the absence of reflections specific to boehmite (see blue reference lines in Figure 2), for longer milling times, boehmite is fully converted by milling. In 1 L steel milling vessel using steel beads, full conversion of boehmite to α-alumina was obtained after ball milling at 1540 rpm for 7 h. The slightly larger milling unit of 2 L volume with the same rotor diameter shows similar conversion times (Figure S3). For the larger scale reaction in an 8 L vessel, the reaction is already complete after 2 h of milling at 950 rpm. The sample taken after 1 h showed some intermediate state. The pattern can be assigned to a mixture of tohdite (5Al_2_O_3_·H_2_O), diaspore (α-AlOOH), and α-alumina. For the 20 L milling chamber, which is able to convert 1 kg of boehmite per batch, the reaction took longer than expected and was not finished after 3 h, although α-alumina was formed partially. From the state of the reaction after 3 h, one could estimate that it might take 5–7 h to reach full conversion to corundum. This reaction, however, could not be completed due to unavailability of the equipment.

The unexpected longer time for milling in the 20 L unit could be attributed to a different density of rotor blades in the milling chamber. The 1 L unit is equipped with 4 blades, the 8 L vessel with 5, and the 20 L milling chamber has 7 rotor blades. The distance between the blades is increasing with increasing milling vessel size. The milling speed is adjusted to the same rotor tip velocity. Since the area covered by a blade in one cycle of the rotor increases with a higher diameter of the rotor, the probability of ball-to-rotor collisions decreases further with increasing scale. Studies by Zoz et al. showed that for the small-scale milling units, it takes milling speeds above 1000 rpm to keep all balls in motion all the time.^29^ From this finding, we expect that the 20 L unit would not move all of the milling balls during the entire milling run. These factors probably all contribute to the longer-than-expected times for full conversion of boehmite to α-alumina for the 20 L milling chamber.

The steel systems with 1 and 8 L volumes seem to keep all balls in motion. Comparing these two different sizes, the result of a faster reaction on a bigger scale appears surprising since one would expect similar reaction times: we kept certain parameters, such as filling volume, ball-to-powder ratio, and temperature, constant. Due to the design of the milling chamber with the horizontal rotor inside and the different rotor blade densities, the maximum ball velocity and collision impact are similar, but velocity distribution and acceleration probability could differ from one chamber size to another. In these terms, the 8 L milling chamber seems to work more efficiently than the smaller 1 L milling chamber to transfer the required energy to drive the conversion to α-alumina.

If the energy transferred in the collisions limits the reaction, then the change in the system size while keeping the other parameters constant should result in a corresponding change in milling time for full conversion. When milling at 950 rpm for the 1 L and 8 L systems, the reaction takes 14 or 2 h, respectively (compare Figure S4). The reaction time changes by a factor of about 7. We assume that the effectiveness of the energy transfer in the system is constant for all scales. Using eq 1, we can calculate the energy transferred for different rotor diameters. The rotor diameter for the 1 L unit is 116 mm,^30^ while the 8 L grinding unit has a diameter of 300 mm. This should correspond to a change in energy transferred to the balls of (300/116)^2^ = 6.7, which corresponds well to the increase in milling time of about 7 from the 8 to 1 L unit.

**Figure 3:**
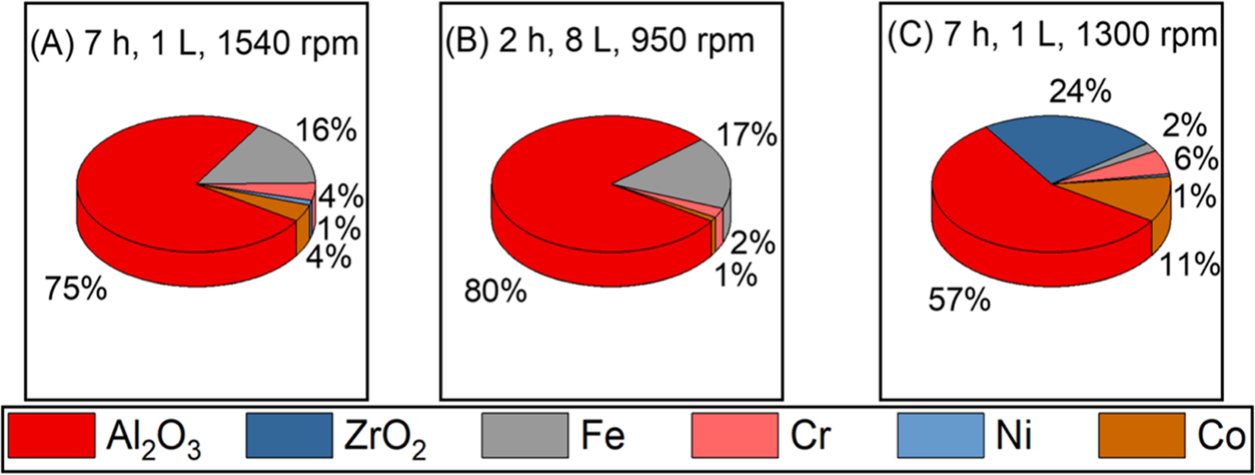
Elemental quantification by SEM-EDX of the alumina samples recovered after ball milling using (A) a 1 L steel vessel and steel balls at 1540 rpm for 7 h, (B) an 8 L steel vessel and steel balls at 950 rpm for 2 h, and (C) a 1 L steel vessel and ZrO_2_ balls at 1300 rpm for 7 h. In all three cases, B:P was 25.

In addition to alumina-related phases (only corundum at full conversion), the XRD patterns show the presence of reflections that can be assigned to iron, which is a contamination due to abrasion of the milling vessel and balls. Even the samples milled for shorter durations show iron reflections due to the abrasion of steel. The scale-dependent change in the conversion time slightly changes the contamination of the product, too. To quantify the amount of contamination, alumina samples recovered from the syntheses were analyzed by scanning electron microscopy-energy dispersive X-ray Spectroscopy (SEM-EDX). The contamination level of 25 wt % for the synthesis in 1 L vessel decreases to 20 wt % for the synthesis in 8 L vessel as shown in Figure 3A,B.

Figure 3A shows EDX analysis of the final alumina sample synthesized in a 1 L steel vessel using steel balls. Besides 75 wt % alumina, 25 wt % steel constituents are detected, specifically 16 wt % iron, 4 wt % chromium and nickel, and 1 wt % of cobalt. The scale-up to an 8 L milling vessel resulted in less contamination as shown in Figure 3B. The SEM-EDX result showed 80 wt % Al_2_O_3_ and in total 20 wt % contamination of steel constituents. Since the iron content is quite high, we decided to change the material of the balls. We decided to use ZrO_2_ balls, a ceramic material with a density close to that of steel (see Table S1). Based on full conversion observed by XRD, the reaction appears to progress much faster with ZrO_2_ balls compared to the reaction with steel milling balls (see Figure S5). It is intriguing to find that ZrO_2_ balls with a lower density than steel balls achieved a faster rate. One potential explanation is that energy is lost in the steel balls due to abrasion and plastic deformation. Moreover, we analyzed the surfaces of zirconia and steel balls (not shown in this manuscript). We found that the surface of zirconia balls is very smooth, while steel ones have much more “dimples”, which could reduce impact since powder in the dimples is less powerfully hit. Therefore, to reach full conversion in a similar time, the milling speed was reduced to 1300 rpm for the case of ZrO_2_ balls. The SEM-EDX quantification results are shown in Figure 3C (right). The lower milling speed also results in a reduction of the contamination by steel constituents, too. The amount of steel contamination was reduced to 20 wt % in total. The fraction of iron is significantly lower, but Cr was increased since the rotor is made from steel with a higher Cr content to increase its hardness. However, now an additional zirconia contamination of 24 wt % is present. Since the ZrO_2_ phase is principally inactive as a catalyst for most applications in catalysis, one may still consider this result as an improvement in lowering the overall level of interfering metal contamination. The results also open room for further optimization using ZrO_2_ balls.

Ball milling of boehmite at different milling speeds was carried out for different milling times to determine the effect of the milling speed on the kinetics of boehmite conversion to corundum. Figure S5 shows the XRD patterns of the powder obtained for different milling speeds at different reaction times, for which pure corundum was detected for the first time. At 1540 rpm the process was completed in 5 h, while 7 h of milling was required for the complete conversion of boehmite to corundum at 1300 rpm. This hints at faster kinetics for boehmite conversion at higher milling speeds. Therefore, we wanted to determine the conversion time per batch of boehmite as a function of milling speed. We performed these kinetic experiments in a slightly larger 2 L milling vessel (similar conversion times were observed for 1 and 2 L vessels, vide supra). The doubling of the vessel’s volume went hand in hand with doubling the batch size and amount of balls to keep the conditions comparable. Various milling speeds were explored for the synthesis of high (specific)-surface-area (HSA) α-alumina with a 2 L milling system.

**Figure 4:**
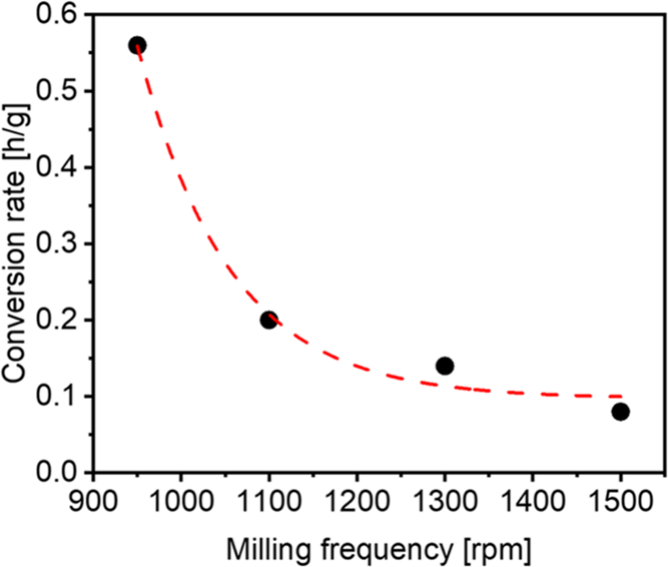
Calculated conversion time per gram of educt for different milling speeds. The trend is emphasized with a red dashed line. All reactions were carried out in the 2 L steel grinding chamber using ZrO_2_ balls until full conversion was reached with a constant B:P ratio.

The time for converting boehmite to α-alumina is normalized by the batch size and plotted against the applied milling speed (Figure 4). The trend of the data points fits to a single exponential decay. For different chamber volumes, a decrease in conversion rate was observed with a decreasing volume (Figure S6). The results imply practical boundaries for the highest and lowest useful milling speeds. Thus, at the lower end of milling speeds, the process will be too slow; in the higher-milling-speed regime, the conditions will result in increased abrasion for a relatively small increase in the rate of conversion (Figure 3).

**Figure 5:**
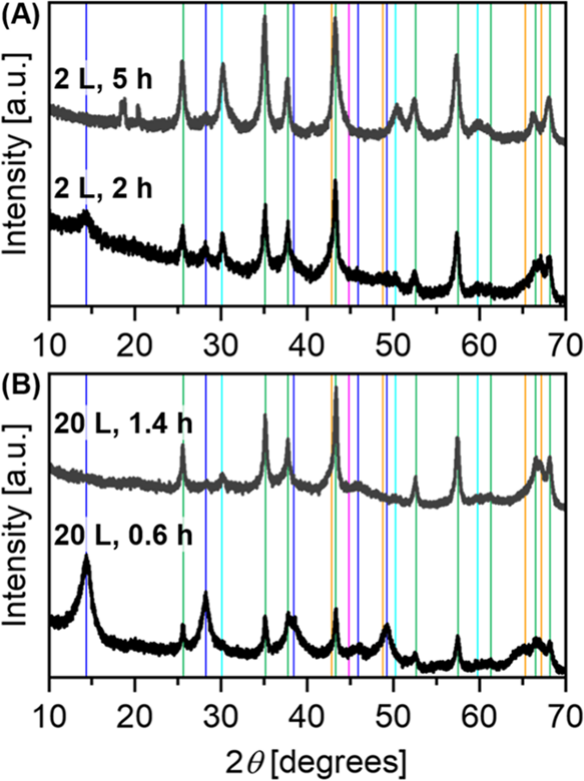
Powder XRD patterns of samples milling in a (A) 2 L and (B) 20 L steel vessel, using ZrO_2_ balls for half and full conversion time. Identified crystalline phases are indicated with vertical lines with color codes: blue: boehmite, green: α-alumina, orange: tohdite, pink: iron, and cyan: ZrO_2_.

The results described above may also suggest that the dehydration of boehmite finishes faster using ZrO_2_ balls than using steel balls. This was independently confirmed by ex situ powder XRD measurements (see Figure S7). Two different experimental designs were selected since the densities of the materials were not the same. On the one hand, the total mass of balls was kept constant, on the other hand, the number of balls and therefore the probability of collisions was kept constant. In both cases, ZrO_2_ balls led to a faster reaction than steel balls. For reactions with ZrO_2_ balls, full conversion was observed in both experiments after 5 h of milling, while for the conversion using steel balls, several XRD reflections were related to intermediates after 5 h of milling. This result further corroborates the better suitability of ZrO_2_ milling balls over those of steel balls. Therefore, ball milling using ZrO_2_ balls in the 20 L milling unit was further investigated, where batch sizes of 1 kg are possible, a scale that is desirable for at least pilot-plant application tests. The phase composition determined by XRD is shown in Figure 5 in contrast to a 2 L vessel reaction at constant B/P ratio, filled vessel volume, and rotor tip velocity.

**Figure 6:**
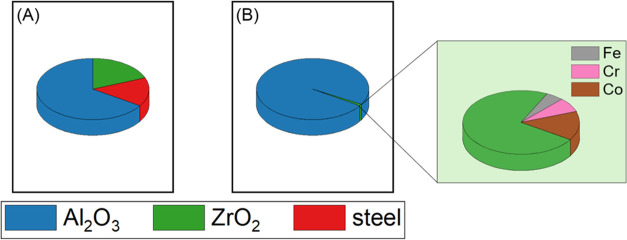
Elemental analysis by SEM-EDX (A) for 2 L and (B) for a 20 L steel milling chamber using ZrO_2_ balls. For (A), 50 g boehmite powder was ball milled in a 2 L vessel using 1.25 kg of milling balls at 1500 rpm. For (B), 1 kg boehmite powder was ball milled in a 20 L vessel using 25 kg of milling balls at the same rotor tip velocity which was achieved at 625 rpm in this case. ICP-OES analysis also confirmed these results for (B).

The first samples (dark gray patterns) were taken after 40% of the full conversion time, i.e., after 2 and 0.6 h, respectively. Residues of boehmite are visible besides the α-alumina phase and minor reflections of ZrO_2_. The residual fraction of boehmite appears to be higher for the sample synthesized on the 20 L scale than for the one derived from the smaller scale reaction, indicating that collision frequency might be lower for the larger vessel, as discussed above. The reaction kinetics with the zirconia balls, however, are much faster than those for the previously used steel balls. Full conversion to α-alumina was reached after 1.4 h, as can be seen from the main reflections in XRD belonging to the product. Besides, reflections of the contamination are almost invisible. The product was also analyzed by SEM-EDX, which showed a significant reduction of the contamination level at increased reaction scale (Figure 6).

Elemental analysis by SEM-EDX and ICP-OES showed a total of only 1.0 wt % contamination for the sample milled in the 20 L vessel. The 1.0 wt % contamination was composed of 0.7 wt % ZrO_2_ and 0.3 wt % of steel constituents from the milling vessel and rotor. This is a very substantial improvement in product properties and synthesis time. Nevertheless, for catalytic applications, even small amounts of transition metals can have detrimental effects. However, the results point toward further reduced contamination levels under optimized conditions at an even bigger scale.

### 2.2 Elimination of Transition Metal Contamination by Optimizing Construction of the Milling Vessel

Given the detrimental effects of transition metal contamination, it was attempted to completely eliminate such contamination. Since leaching out the transition metals, similar to the work done previously on a smaller scale in WC equipment, did not work for steel contamination,^3^ it was decided to avoid the contact of the alumina to steel as much as possible during the milling process. The scale-up process was therefore studied by using an all-ceramic unit. In such a system, plates of Si_3_N_4_ line the inner walls of the milling chamber, and the rotor blades are made of Si_3_N_4_ as well (Figure 1C). Si_3_N_4_ of two different qualities, produced using two different sintering additives and sintering processes, were used. We chose Si_3_N_4_ out of the possible materials since WC appeared to be too expensive, and it is often combined with cobalt, which should also be avoided. Other ceramic materials, in particular corundum and ZrO_2_, were excluded, since already published works had suggested that they would most probably be unsuitable.^31,32^ Moreover, Si_3_N_4_ milling equipment was commercially available from the manufacturer of the Simoloyer.

**Figure 7:**
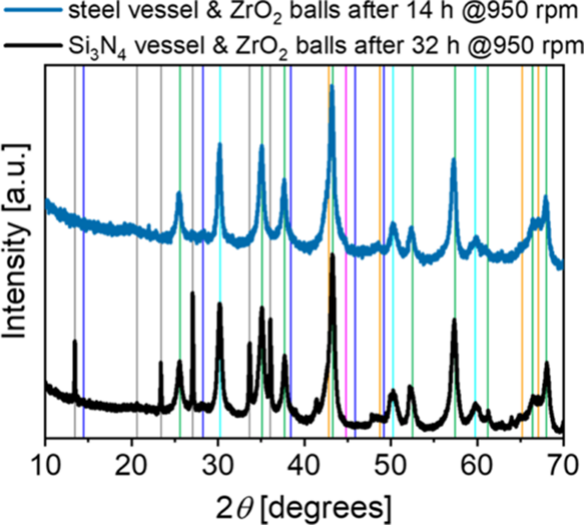
Powder XRD patterns of products after milling in the steel or Si_3_N_4_ vessel using ZrO_2_ balls in both cases. Identified crystalline phases are indicated with vertical lines with color code: blue: boehmite, green: α-alumina, orange: tohdite, pink: iron, cyan: ZrO_2_, and gray: Si_3_N_4_. Milling conditions: 1 L steel or Si_3_N_4_ lined vessel, 625 g zirconia milling balls (⌀ 5 mm), 25 g boehmite, 950 rpm.

The manufacturer recommended 1000 rpm as the maximum milling speed for the ceramic milling unit. In order to be on the safe side, a slightly lower milling speed, i.e., 950 rpm, was used. This, however, led to longer milling times to obtain full boehmite conversion to α-Al_2_O_3_. We also explored a milling speed of 875 rpm and found that it is the minimum milling speed for conversion within a reasonable time period (Figure S8). Therefore, we selected 950 rpm since the small difference reduces the time for full conversion but does not significantly affect the contamination level. The crystalline phases present in the final sample were identified by powder XRD (Figure 7). The mechanochemical conversion of boehmite to α-alumina was found to be slower using the ceramic unit than the steel chamber (included for comparison) with the same mass of ZrO_2_ balls. In both cases, full conversion to α-alumina was observed after 14 and 32 h, respectively, in steel and silicon nitride (Si_3_N_4_) vessels. The reaction time for the latter is relatively long. This might be caused by the noneven surface inside the Si_3_N_4_ vessel due to the high number of individual plates. This may change the ball movement, in addition, powder may be trapped in the grooves between the tiles for some time. Both factors may slow the conversion (Figure 7). The sharp reflections present in the XRD pattern of powder milled using a Si_3_N_4_ vessel and ZrO_2_ balls are due to Si_3_N_4_ abrasion. Since contamination from Fe, Cr, Ni, and Co cannot be tolerated for most applications, the ceramic-lined system is considered as the best option for this process, in spite of the contamination by Si_3_N_4_ and ZrO_2_.^33^

**Figure 8:**
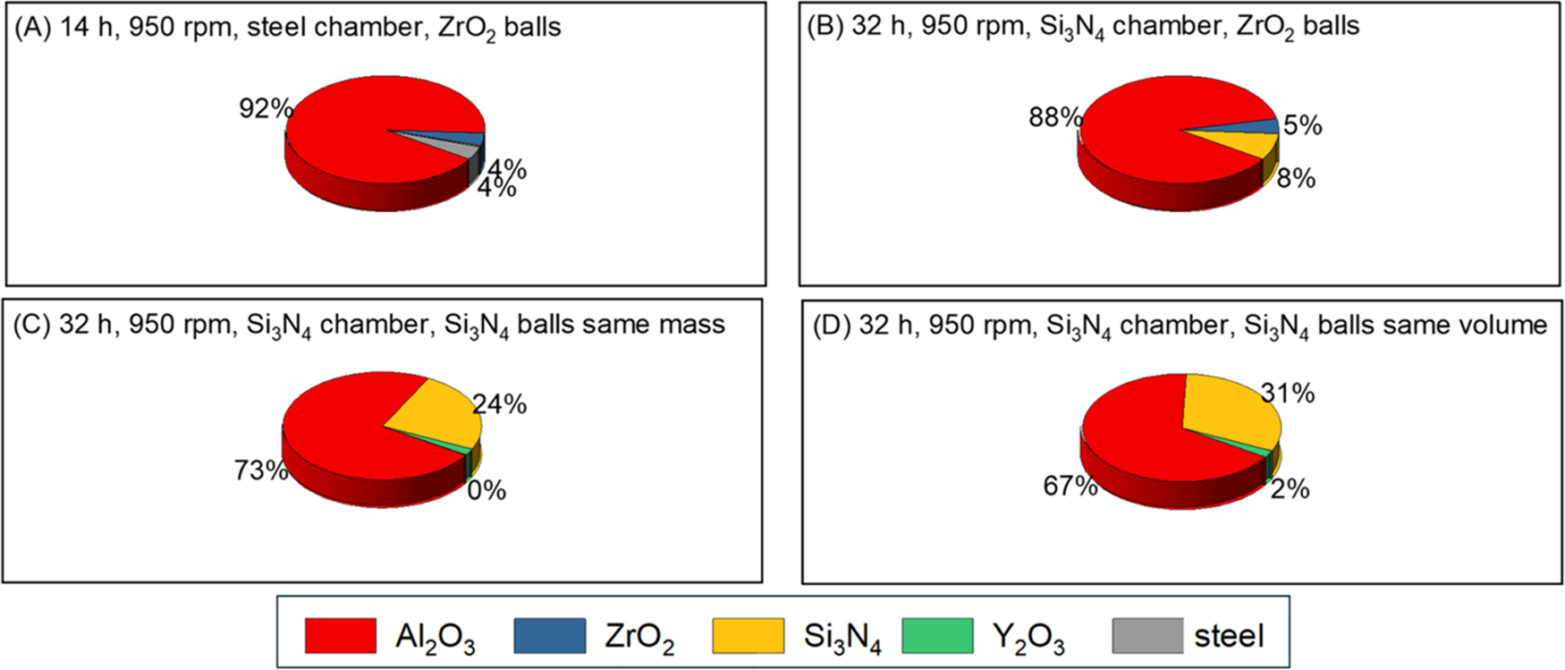
Elemental analysis by SEM-EDX of products recovered after (A) 14 h and (B–D) 32 h of milling at 950 rpm using ZrO_2_ or Si_3_N_4_ balls of the same mass.

SEM-EDX analysis of these samples showed low levels of contamination due to abrasion (Figure 8), which might be due to the low milling speed applied. For the steel vessel with ZrO_2_ milling balls, the contamination was reduced to 4 wt % ZrO_2_ and 4 wt % steel constituents (Figure 8A). For the Si_3_N_4_ lined vessel with ZrO_2_ balls, 5 and 8 wt % contamination of ZrO_2_ and Si_3_N_4_, respectively, were observed (Figure 8B). These results show that contamination can be restricted to ceramic materials in such mills.

**Figure 9:**
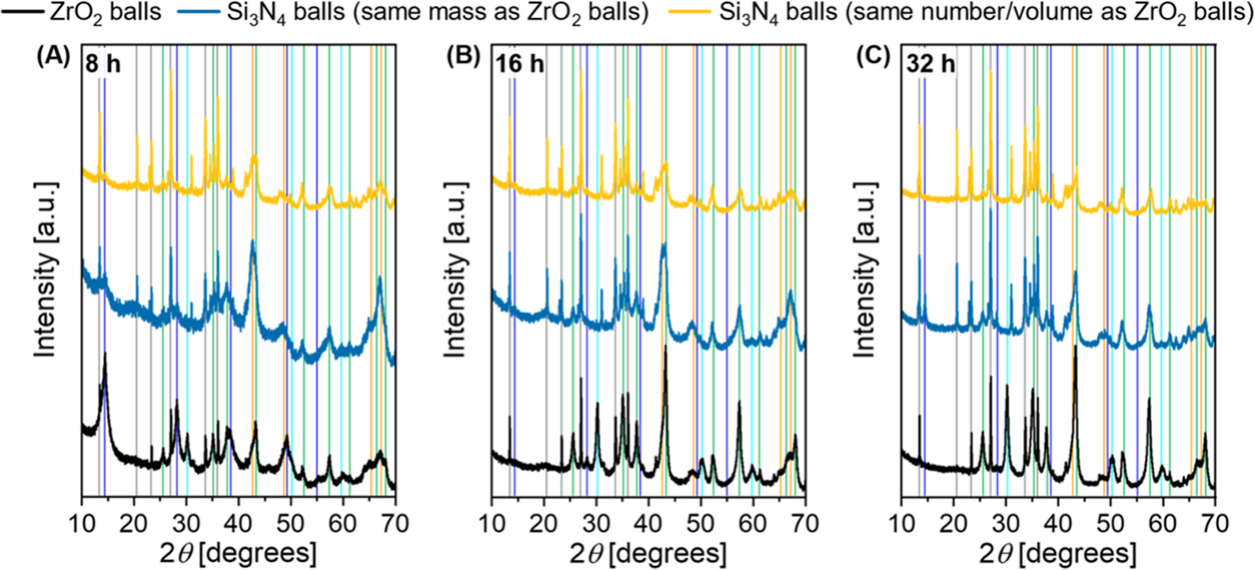
Powder XRD patterns measured after 8, 16, and 32 h for a Si_3_N_4_ lined vessel using ZrO_2_ balls, Si_3_N_4_ balls of the same mass or volume as for ZrO_2_. Identified crystalline phases are indicated with vertical lines with color code: blue: boehmite, green: α-alumina, orange: tohdite, pink: iron, cyan: ZrO_2_, and gray: Si_3_N_4_. Milling conditions: 1 L Si_3_N_4_ lined vessel, 625 g zirconia milling balls (yellow) or Si_3_N_4_ (blue) (⌀ 5 mm) or 487 g Si_3_N_4_ balls, 25 g boehmite, 950 rpm.

To investigate if the contamination can be limited to one type of material, Si_3_N_4_ balls were used for ball milling of boehmite in a Si_3_N_4_ milling vessel. Since Si_3_N_4_ has a lower density than steel and zirconia, longer milling times were expected. The results are summarized in Figure 9. Powder XRD patterns were collected after 8, 16, and 32 h of milling using either ZrO_2_ balls or Si_3_N_4_ balls of the same diameter. For Si_3_N_4_ balls, both, reactions with the same mass as ZrO_2_ balls and with the same volume of balls, were studied. The longer the reaction times, the more intense the reflections related to the α-alumina phase become. Furthermore, the Si_3_N_4_ and ZrO_2_ reflections gain intensity, while reflections of boehmite are reduced in intensity.

The ball material density differs significantly, 6.0 g/cm^3^ for zirconia and 3.2 g/cm^3^ for Si_3_N_4_ (see Table S1). Therefore, keeping the same ball-to-powder weight ratio leads to a significantly higher number of balls used for Si_3_N_4_. Independent of the ball material, the conversion to α-alumina was completed after 32 h of milling at 950 rpm in the Si_3_N_4_ lined 1 L vessel for the same number of milling balls. However, with the same mass of Si_3_N_4_ balls, the conversion was not complete after 32 h of milling. At the same time, the change to Si_3_N_4_ as the ball material leads to a significant increase of Si_3_N_4_ contamination (Figure 8C). Even if the number of collisions per time is increased compared with using ZrO_2_ balls, the collisions involving the Si_3_N_4_ balls seem to be significantly less effective. Another factor is the overall filling of the milling vessel. With the less dense Si_3_N_4_, the vessel is rather full, so that balls move less freely and undergo more ball-to-ball collisions, which could reduce the effectiveness of reactive collisions and overall lead to increased abrasion. One should note that also Y_2_O_3_ was detected as an impurity, which is added to the Si_3_N_4_ during production as a sintering aid (Figure 8C) and thus also is abraded. Moreover, it turned out that the Si_3_N_4_ balls used were not stable for more than one synthesis batch (see Figure S9, the surface of the balls is irregular and pitted after one run). Thus, if silicon nitride should be used as the material for the milling balls, it would be necessary to develop a sintering process for the production of more stable Si_3_N_4_ balls, perhaps along similar lines as the production of higher-quality Si_3_N_4_ used for the tiles lining the milling vessel.

Overall, the Si_3_N_4_ lined milling vessel with ZrO_2_ milling balls seems to be the best combination for the mechanochemical dehydration of boehmite to α-alumina, as it allows the production of high-surface-area corundum with no detrimental metal contamination.

### 2.3 Properties of Corundum Obtained in Scale-Up Experiments

**Figure 10:**
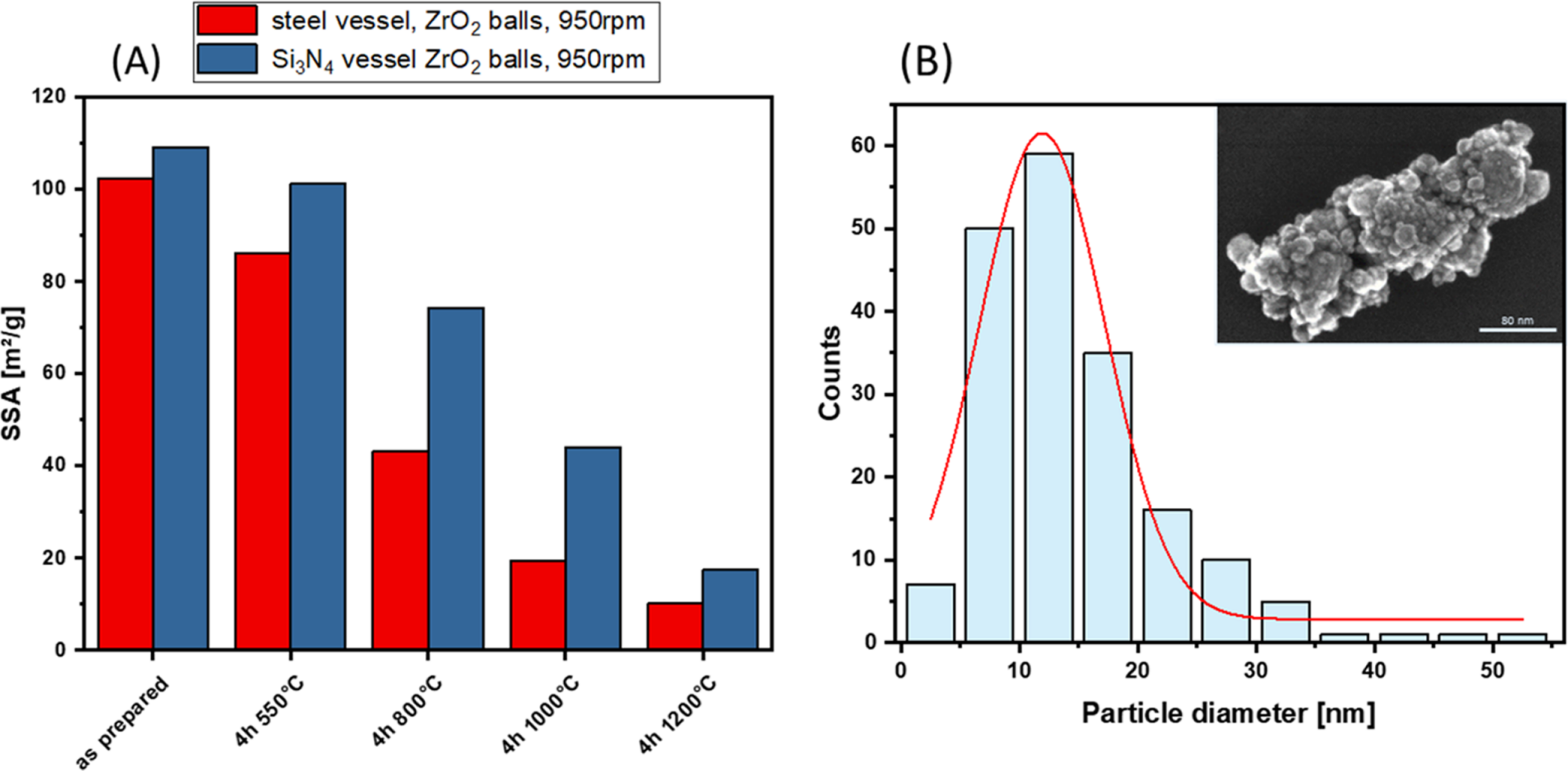
(A) Specific surface area of synthesized α-alumina using different vessel materials and ZrO_2_ balls in as prepared form and after heat treatment at different temperatures for 4 h. (B) α-Alumina particle size distribution determined from TEM images after synthesis at 950 rpm using ZrO_2_ balls in the Si_3_N_4_ lined vessel.

The specific surface areas (SSA) of samples after full conversion of boehmite to α-alumina are summarized in (Table 1). The α-alumina from the scaled-up synthesis generally seems rather similar to that of the samples obtained from laboratory-scale experiments. Specific surface areas of up to 110 m^2^/g (Table 1) and average particle sizes of 13 nm were obtained under optimized conditions (see Figure 10B). With Si_3_N_4_ balls, the dehydration cannot be driven to α-alumina with high surface area. The surface area of the obtained product is only around 34 m^2^/g.

**Table 1:** Properties of α-Alumina Formed in Different Scale-Up Experiments^a^

material “vessel-balls”	milling speed (rpm)	time for full conversion (h)	batch size (g)	*S*_BET_ (m^2^/g)	amount of milling balls (g)
SN–SN	950	32	25	19	487
SN–SN	950	32	25	34	625
SN–ZR	950	32	25	109	625
SS–ZR	950	14	25	108	625
SS–ZR	1500	7	50	43	1250
SS–ZR	1540	7	50	87	1250
SS–SS	1750	7	50	87	1250

a Abbreviations: SS stainless steel; ZR for ZrO_2_, and SN for Si_3_N_4_.

For applications as catalysts or support materials, the HSA α-alumina will be exposed to high temperatures during catalysis. Thus, the stability of the surface area at temperatures up to 1200 °C was studied.

For the alumina synthesized in the ceramic vessel with zirconia balls, a high SSA of about 110 m^2^/g was achieved. The surface area for this sample remains more stable upon heat treatment at various temperatures compared to the partially steel-contaminated surface area, which had been obtained in the steel vessel using ZrO_2_ balls (Figure 10). The SSA of the materials milled in the ceramic unit is consistently higher after treatment at different temperatures. After calcination at 1000 °C, the remaining SSA is almost twice as high for the material from the ceramic-lined vessel compared to the steel vessel, and this is rather similar for treatment at 1200 °C. SSA of the corundum obtained in the ceramic vessel is slightly below 20 m^2^/g, which is quite remarkable for a material treated at such harsh conditions. This makes the production of alumina by ball milling with zirconia balls even more interesting if the desired application is as catalyst support, for instance, in automotive exhaust catalysts. The contamination seems to stabilize the SSA of the material.

Therefore, the use of ZrO_2_ balls in a vessel lined with Si_3_N_4_ ceramics, or potentially in a complete zirconia system, seems highly interesting. Our future work will explore the synthesis in a milling chamber completely manufactured from zirconia. A promising design is currently under construction.

## 3 Conclusions

The mechanochemical synthesis of high-surface-area α-alumina from boehmite on a scale of up to 1 kg per batch was developed using Simoloyer ball mills. Various parameters were optimized such as materials of milling chambers (steel and Si_3_N_4_), materials of milling balls (steel, ZrO_2_, Si_3_N_4_), amount of milling balls and powder (to vary B:P ratio), impact energy or milling speed, and milling time. Boehmite conversion to corundum was studied on scales from 1 to 20 L milling vessel volume, enabling ball milling of 50 g to 1 kg boehmite powder. The impact of the milling balls and the transfer of energy to the powder via collisions are essential for the reaction. The impact is also dependent on the materials of the milling chamber, balls (and thus their density), and the applied speed of ball milling. Also, the problem of transition metal contamination for possible applications was tackled. The results show that the contamination with transition metals can be avoided by operating the reaction in a ceramic system composed of a milling chamber lined with Si_3_N_4_ and using ZrO_2_ milling balls for optimal impact. This combination also results in phase pure material with a high surface area. Future work is focused on developing larger all-ceramic systems with milling chambers homogeneously lined with ceramic materials instead of individual plates. The results of this study overall suggest that the production of commercially relevant amounts of high-surface-area corundum is possible. Given the fact that the Simoloyer mill type is available up to 900 L chamber volume, an increase in batch size up to 50 kg of material appears possible, which would allow the production of several ten tons of material per year in a single mill.

## 4 Experimental Section

Boehmite (Pural 200) was received from Sasol for research purposes and used as received. Milling balls of 5 mm diameter made of steel and zirconia were obtained from Zoz GmbH. Milling balls of Si_3_N_4_ were used as received from MSE supplies. For experiments using 1 and 2 L vessels, the CM01 unit procured from Zoz GmbH was used, whereas for experiments using 8 and 20 L vessels, the CM08 and CM20 Simoloyer operated at Zoz GmbH were utilized. Milling chambers of 1, 2, 8, and 20 L made of stainless steel and a 1 L Si_3_N_4_ lined chamber were used as reaction environment.

In ref (34), the operation mode, variations in size, and lining material for different models of the Simoloyer are described in detail. In general, one can simplify Simoloyer as a high-kinetic-energy rotary ball mill. The rotor on a horizontal drive shaft is moved at high speed. Its blades accelerate the balls to drive the desired reaction. Double-walled grinding vessels with inner volumes of 1, 2, and 8 L made of stainless steel (AISI 304) were used on CM01 and CM08, respectively. The rotors for stainless steel vessels were also made of stainless steel (AISI 304). The tips of the rotor blades were made of Stellite (Co-based alloy) for high stability. A 1 L stainless steel vessel, the inside wall of which is lined with Si_3_N_4_ plates, was used as well. It also has a rotor, blades, and tips of blades all made of Si_3_N_4_ or lined with Si_3_N_4_. This system is hereafter referred to as the Si_3_N_4_ vessel and the whole unit as the Si_3_N_4_ unit. Grinding media made of stainless steel (AISI 50100; ⌀ = 4.76 mm), yttria-stabilized zirconia (YSZ; ⌀ = 5.00 mm), or Si_3_N_4_ (⌀ = 5.00 mm) were used. In a typical experiment, the milling vessel is loaded with milling balls and boehmite powder (Sasol, Pural 200) in a ball-to-powder weight ratio (BPR) of 25, unless otherwise stated. Milling speeds of up to 1750 rpm and up to 950 rpm were applied on a CM01 steel vessel and CM08 or CM01 Si_3_N_4_ units, respectively. Throughout the experiments, the temperature of the double-walled milling vessel was maintained at ca. 20 °C by circulating cold water through it. The actual temperature at the inner wall, which was continuously recorded by a thermocouple, did not exceed 45 °C in any of the experiments reported herein. During milling operation, power consumption was also recorded for different capacities and milling speeds studied. Samples were collected at different time intervals by pausing the milling operation.

The commercially available Si_3_N_4_ clad milling vessels were found to be unsuitable for the synthesis of high-surface-area corundum. After only 7 h of operation, the tiles showed visible abrasion; moreover, in the vicinity of the opening of the vessel (to allow sampling), some of the tiles had detached from the walls and were broken; fragments of about 1 cm^2^ in size could be isolated from the milled product. These problems were solved by two measures: (i) the tiles were replaced by new tiles supplied by IKTS Dresden, which had been produced by a special high-temperature sintering process under argon atmosphere. These tiles also have a higher yttria content. They were glued to the wall of the milling vessel with the same epoxy glue also used for the original tiles. (ii) The origin of the detachment of the tiles around the sampling opening was found in insufficient cooling in this region, which led to softening of the glue. This region of the vessel was therefore modified by an additional cooling unit made from an aluminum block in this part of the vessel. These two modifications allowed operation of the mill for at least 32 h without visible damage to the Si_3_N_4_ cladding. Besides, the addition of new tiles did not significantly reduce the inner volume of the vessel.

For specific surface area stability testing samples (500 mg) were heated up using a heating rate of 5°/min, afterward the temperature was kept for 4 h. For 500 and 800 °C, the Nabertherm LV15/11/P320 was used. Higher temperatures of 1000, 1100, and 1200 °C were realized using the Nabertherm L08/14 furnace.

The obtained powders after ball milling under various conditions were analyzed by powder XRD, N_2_ sorption, SEM-EDX, ICP-OES, and TEM analysis.

Powder XRD patterns were collected using Bragg–Brentano geometry instruments; either X’Pert, Rigaku, or Stoe STADI P diffractometer were used. All machines were operated with Cu Kα radiation. The patterns were collected from 10 to 75° 2θ. The crystalline phases present in the sample were identified by using the PDF-2 2013 database. The following PDF files were used to assign the phases: ICDD PDF 21-1307 for boehmite (γ-AlOOH), ICDD PDF 22-1119 for tohdite (5Al_2_O_3_·H_2_O), ICDD PDF 46-1212 for α-Al_2_O_3_, ICDD PDF 06-0696 for Fe, ICDD PDF 00-049-1642 for ZrO_2_, and ICDD PDF 00-033-1160 for Si_3_N_4_.

N_2_ sorption was carried out using a 3Flex instrument from Micromeritics. The samples (250–450 mg) were degassed in a vacuum at 140 °C for 12 h prior to the measurement. The specific surface area (*S*_BET_) was calculated from the adsorption data measured at −196 °C in the relative pressure range of 0.05–0.3 using the Brunauer–Emmett–Teller (BET) method.

For SEM-EDX spectra, the tabletop instrument Hitachi TM3030 plus was used. It was equipped with an additional Xplore Compact 30 detector from Oxford Instruments. The acceleration voltage was set to 15 kV and the detection mode was set to EDX during the measurement. The samples were prepared by sprinkling dry specimens on a Carbon film.

For ICP-OES 30–50 mg of the sample was dissolved in concentrated HCl-solution together with one-fourth of HNO_3_-solution and heated to 180 °C for 10 min right afterward in a microwave. The solution was diluted with mQ-water and measured in an ICP-OES setup using an argon plasma for ionization and optical detectors for the required wavelength according to the desired elements. For quantification three external standard solutions were used per element, the sample signal was compared to the signals of known corresponding solutions.

For determination of the particle size, TEM images were taken at 200 kV acceleration voltage with the Hitachi HD 7200, in Cs-corrected dedicated STEM mode and using a cold field emission gun (FEG). Images of the secondary electron mode in the bright-field configuration were used for particle size determination.

## Supporting Information Available

Additional characterization data (PDF)
