# Supplementary material for: Scale-Up of Nanocorundum Synthesis by Mechanochemical Dehydration of Boehmite
Source: Ind Eng Chem Res. 2025 Jan 13;64(3):1577–86. doi: 10.1021/acs.iecr.4c03537 (PMC11760161; doi:10.1021/acs.iecr.4c03537)
Supplement: Supplementary file 1 — ie4c03537_si_001.pdf [file ie4c03537_si_001.pdf]

Supporting Information to

Scale-up of nano-corundum synthesis by mechanochemical dehydration of boehmite

Sarah Triller,[a] Amol P. Amrute,[a,b] and Ferdi Schüth[a]

[a]Max-Planck-Institut für Kohlenforschung, Kaiser-Wilhelm-Platz 1, D-45470 Mülheim an der Ruhr, Germany.

[b]present address: Institute of Sustainability for Chemicals, Energy and Environment (ISCE^2), Agency for Science, Technology and Research (A*STAR), 1 Pesek Road, Jurong Island, 627833, Singapore.

Table S1. Properties of the milling materials.

Material	Density [g/cm^3]
Steel	7.83
ZrO_2	6.0
Si_3N_4	3.24

In the following sections, different kinds of mechanical force interactions will be discussed. Therefore, the term ball-to-ball collision will be used for cases when two moving balls collide with each other while powder is trapped between them. In addition, the term ball-to-wall collision will be used similarly, referring to a collision of a milling ball and the chamber wall with powder trapped between the surfaces.

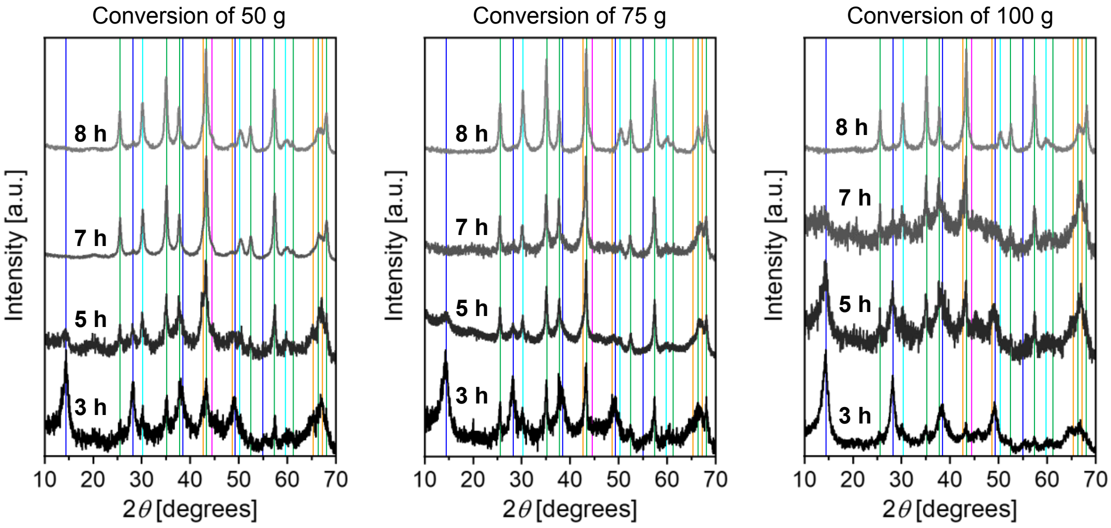

Figure S1. Powder XRD patterns of boehmite samples after ball milling for different durations of different amounts of boehmite (50, 75, 100 g) under the same milling parameters using a 2-L grinding unit. Milling conditions: 2 L steel vessel, 1250 g zirconia milling balls (∅ 5 mm), 50-100 g boehmite, 1300 rpm. Positions of single-phase reflections are indicated with vertical lines of color code: blue: boehmite, green: α-alumina, orange: tohdite, pink: iron, and cyan: ZrO_2.

We used the 2-Liter steel unit to study the effect of volume filled and ball-to-powder ratio on the boehmite conversion by ball milling at the several 10-gram scale. Keeping all milling

conditions the same, we changed the amount of boehmite used for milling. The reaction progress is measured by analyzing powders milled for different times (Figure S1). Changing the amount of powder in the range of 50-100 g did not significantly alter the filled volume, since this is dominated by the balls. For 50, 75, and 100 g of educt, the conversion to α-alumina is completed after 8 h using 1250 g of ZrO_2 balls and milling at 1300 rpm. By comparing the samples after 5 h of milling, it can be seen that the first vanishing boehmite reflection at 14°2θ is still present for the higher loadings. This suggests a little slower conversion rate for higher boehmite amounts. Nevertheless, this difference gets smaller after 7 h of milling and the progress of the reaction with 75 g of boehmite is comparable to the one with 50 g. Notably, the increase in the amount of powder is inversely proportional to the ball-to-powder (BPR) mass ratio. The B:P ratios for amounts of 50, 75, and 100 g are 25, 17, and 12.5, respectively. Changing the amount of balls instead will change the filled volume in addition to the change in B:P ratio. The related change in the dehydration process of boehmite is visualized in Figure S2 using XRD results.

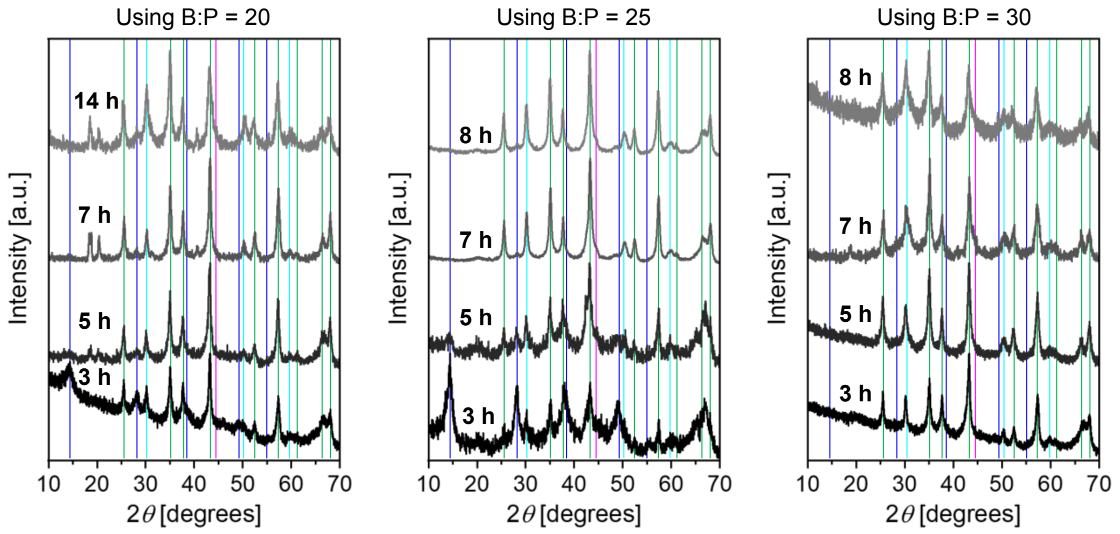

Figure S2. Powder XRD patterns of samples after 3, 5, and 7 h of milling and the final product pattern at variable ball-to-powder weight ratio in the 2-Liter grinding unit at 1300 rpm. B:P is

varied at a fixed powder amount of 50 g and by varying the weight of balls. Milling conditions: 2 L steel vessel, 1000-1500 g zirconia milling balls (∅ 5 mm), 50 g boehmite, 1300 rpm. Positions of single-phase reflections are indicated with vertical lines of color code blue: boehmite, green: α-alumina, pink: iron, and cyan: ZrO_2.

By changing the filling ratio with the B:P ratio, the conversion rate is much more strongly affected than before. Upon decreasing the B:P to 20, 14 h of milling was required to achieve full conversion, in contrast to 8 h (Figure S1). Upon increasing the B:P ratio to 30, 8 h of milling was still required to reach the full conversion, which is similar to that required for 17.5-25 B:P in Figure S1. The case of increased ball amounts is also not desirable since the change seems to make ball-to-ball or ball-to-wall collisions without powder being present (where impact is felt by balls or ball and wall) more likely than such collisions with powder trapped between balls or ball and wall (where impact is felt by powder). The contamination level for these samples from zirconia balls does not increase, even if the number of collisions is increased. This emphasizes the high stability of the ZrO2 balls and the main origin of the contamination by the ball-to-wall collisions (with formed corundum powder being trapped between the ball and wall) due to the formation of the very hard alumina.[1]

Further lowering the B:P ratio to 20, the filled volume drops by 20%. Therefore, the probability of ball-to-wall or ball-to-ball collisions, both trapping powder during the collision, decreases and the reaction takes longer (see ref.2). The decreased filling level is the crucial effect here since changes in the B:P ratio with an essentially unchanged filling level (SI Figure 1) do not result in substantially changed conversion times.

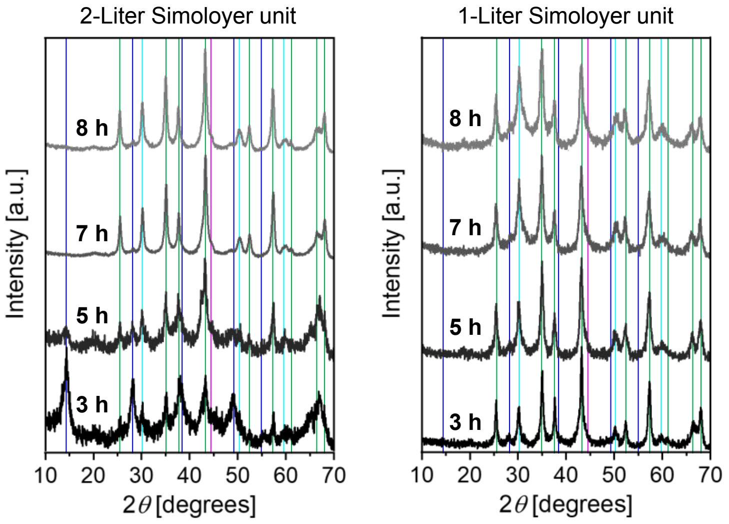

Figure S3. Powder XRD patterns of samples after 3, 5, 7, and 8 h of ball milling using 1-Liter or 2-Liter vessel. Milling conditions: 1 or 2 L steel vessel, 1250 g zirconia milling balls (∅ 5 mm), 50 g boehmite, 1300 rpm. Positions of single-phase reflections are indicated with vertical lines of color code: blue: boehmite, green: α-alumina, pink: iron, and cyan: ZrO_2.

Figure S3 compares the conversion of boehmite using 1-Liter and 2-Liter vessels on the Simoloyer® CM01 unit. The progress was monitored by XRD analysis of samples after 3, 5, 7, and 8 h of milling. Besides the vessel sizes, all other parameters were kept constant. It can be seen from the XRD patterns that the conversion in the larger unit is slower at the start compared to its 1 L analogue; however, the formation of the final α-alumina product requires the same time (7 h). Since both rotors have the same diameter and the 2-L unit has one blade more than the 1-L unit, the impact is the same in both cases. The impact seems to be more important than other parameters since ball and powder are much more 'diluted' in the case of the larger unit, but this does not change the final output. The different collision frequency in both systems seems to influence the induction of the reaction stronger than the latter process. It should be noted here that even though the filling volume is reduced for the 2-L vessel case compared to the 1-L case (SI Figure S3), it did not significantly affect the conversion of boehmite to corundum, except the initial induction period. This contrasting behavior, compared to the case above (SI Figure S2), where lowering the filling ratio by reducing the amount of milling balls affected the conversion rate significantly, may be explained by the fact that in this case, the B:P ratio was unchanged which could still provide a similar number of collisions and a similar impact. Whereas in the above case, the number of balls was reduced (SI Figure S2), which may result in a lower number of collisions and thus in a reduction of the overall impact on the powder, leading to a longer milling time to achieve full conversion of boehmite to corundum.

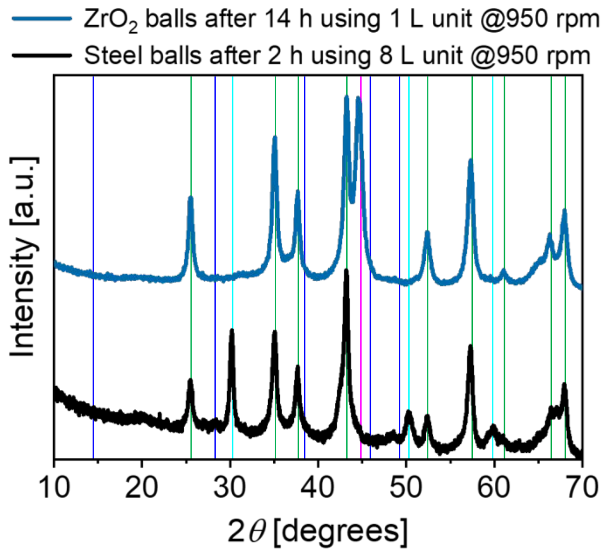

Figure S4. Powder XRD patterns boehmite samples after its full conversion to α-alumina by ball milling in 1 L vessel using ZrO_2 balls or in 8 L milling chamber using steel balls at 950 rpm. The reaction was faster in the 8 L vessel. Positions of single-phase reflections are indicated with vertical lines of color code: blue: boehmite, green: α-alumina, pink: iron, and cyan: ZrO_2.

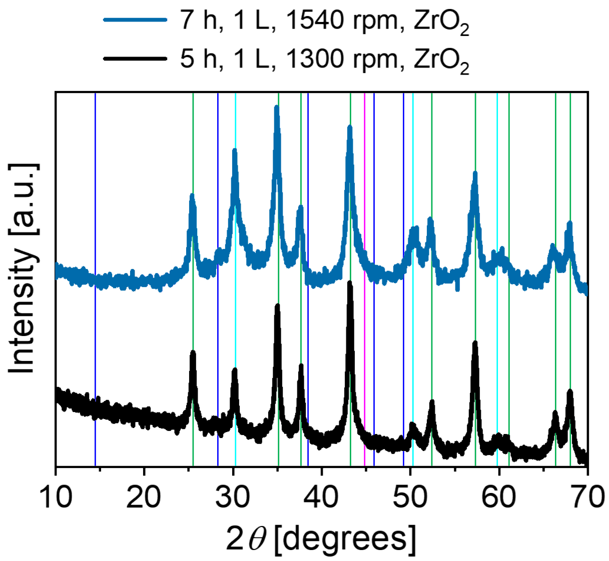

Figure S5. Powder XRD patterns of samples after milling in 1 L vessel at 1300 rpm and 1500 rpm using ZrO_2 balls. Milling conditions: 1 L steel vessel, 1250 g zirconia milling balls (∅ 5 mm), 50 g boehmite, 1500 or 1300 rpm. Positions of single-phase reflections are indicated with vertical lines of color code: blue: boehmite, green: α-alumina, pink: iron, and cyan: ZrO_2.

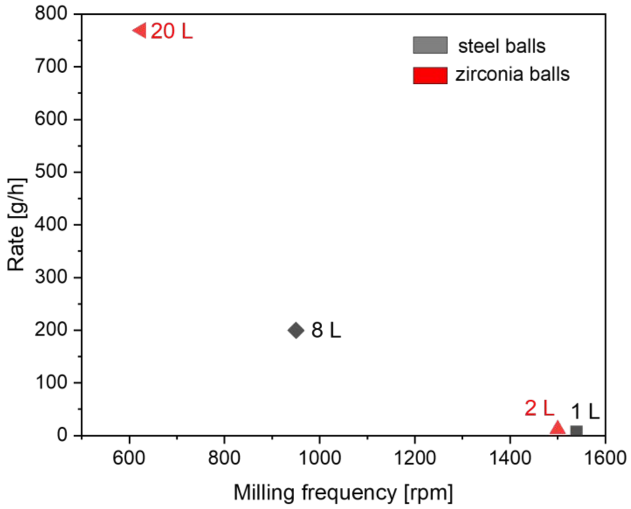

Figure S6. Conversion rates for the same rotor tip velocity in 1, 2, 8, and 20 L milling chambers, reaction carried out with same B:P ratio and filling ratio, either using steel or ZrO_2 balls of 5 mm diameter. The milling frequency decreases with increasing reaction scale for the same rotor tip velocity.

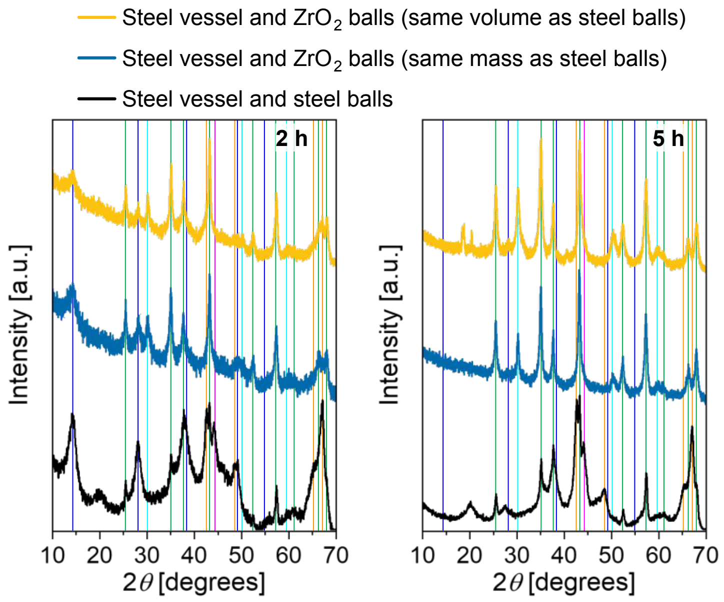

Figure S7. Powder XRD patterns of samples taken after 2 and 5 h of milling at 1300 rpm in a steel vessel using either steel balls or ZrO_2 balls of the same mass or volume as for steel. Positions of reflections are indicated at the bottom axis with vertical lines of color code: blue: boehmite, green: α-alumina, orange: tohdite, pink: iron, and cyan: ZrO_2. Milling conditions: 2L steel vessel, 1250 g steel (black) or zirconia milling balls (blue) of ∅ =5 mm or 922 g zirconia balls (yellow), 50 g boehmite, 1480 to 1500 rpm.

Figure S7 shows the progress of the dehydrative phase transformation of boehmite to α-alumina using different amounts and materials for the balls in a steel vessel. The black, blue, and yellow lines are the XRD patterns of samples after 2 or 5 h, milled with steel and zirconia balls of the same mass and the same volume of zirconia balls, respectively. α-Alumina phase reflections are more prominent for samples milled for 5 h than for 2 h (marked with green lines), while the boehmite-related reflections vanish after this prolonged time. For all patterns, additional reflections that are related to the ball and vessel material are also visible.

From XRD patterns in Figure S7 for 2 h milled or 5 h milled samples, it can be seen that the nature of milling ball materials also seems to influence the boehmite conversion to corundum, with zirconia appearing to be more effective.

Figure S8 shows results after 10 h of ball milling of 25 g of boehmite with ZrO_2 balls (625 g) in a Si_3N_4 lined milling chamber at different frequencies from 500-950 rpm. The lowest frequency required to form α-Al_2O_3 is 875 rpm and therefore, we opted for 950 rpm for studying the milling process in the Si_3N_4 chamber (see main manuscript Section 2.2).

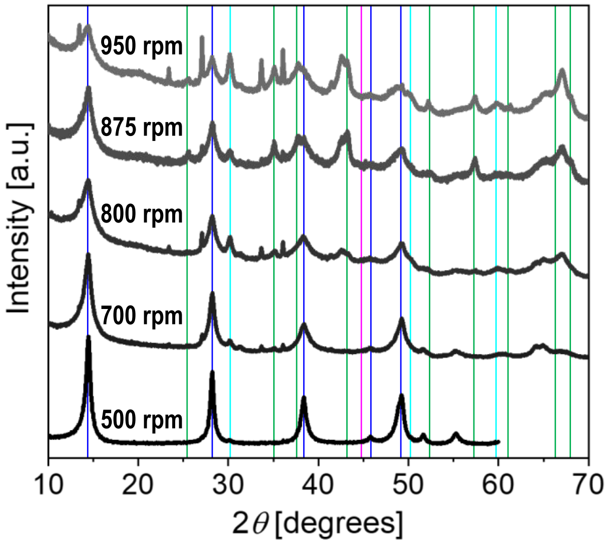

Figure S8. Conversion state after 10 h of milling boehmite (25g) with ZrO_2 balls (625 g) at various frequencies (500 rpm to 950 rpm) using the Si_3N_4 lined milling chamber. Positions of single-phase reflections are indicated with vertical lines of color code: blue: boehmite, green: α-alumina, pink: iron, and cyan: ZrO_2.

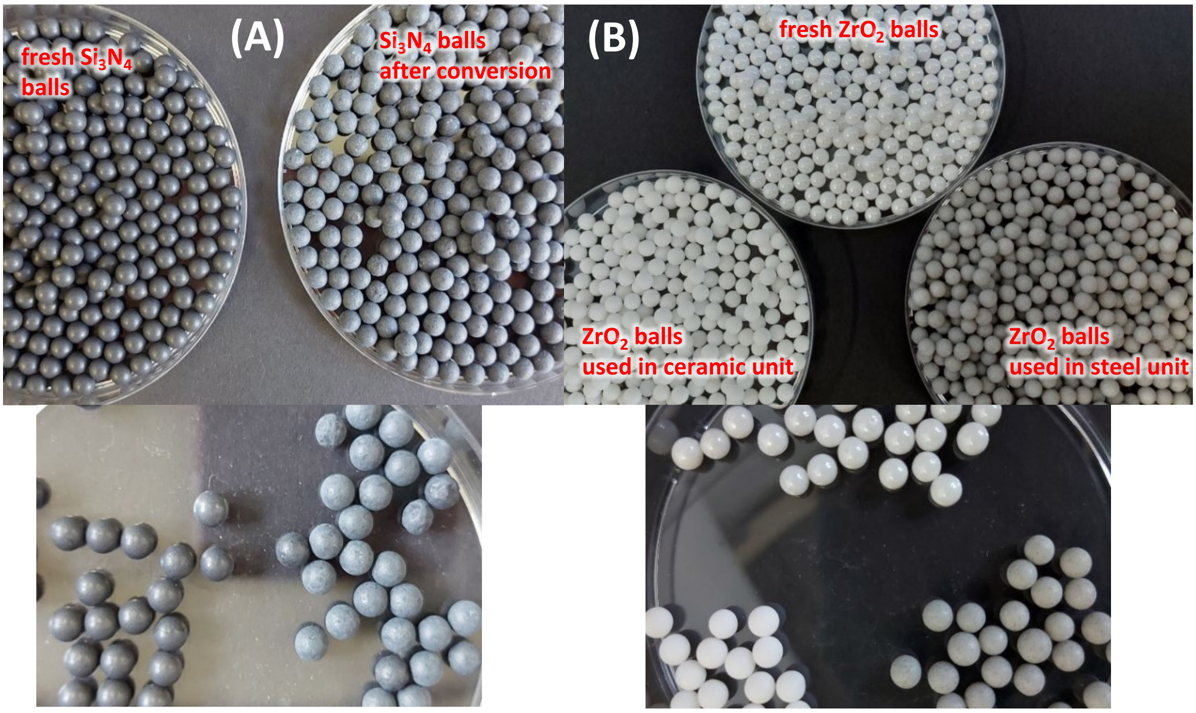

Figure S9. Ceramic milling balls before and after use; (A) Si_3N_4 balls left before and right side after synthesis of alumina, (B) ZrO_2 balls top: before, after synthesis in left: Si_3N_4 unit and right: steel unit. The bottom part shows close-ups from the balls. Milling conditions: 1 L steel vessel, 625 g milling balls (∅ 5 mm) Si_3N_4 (A) or zirconia (B), 25 g boehmite, 950 rpm until full conversion was reached.

Figure S9 shows photographs of milling balls before and after ball milling boehmite. They show strong abrasion of Si_3N_4 balls after the mechanochemical conversion. It can be seen that the change for the Si_3N_4 balls, in (A) is more dominant than for the zirconia balls (B) after running a conversion in the 1-Liter milling units for both materials. The steel abrasion leads to a greyish appearance of the ZrO_2 balls after the reaction, in the fully ceramic systems the balls remain white, only getting less shiny during milling, which indicates some roughening of the surface.

1. Prokhorov, I. Y.; Akimov, G. Y.; Timchenko, V. M., Stability of structural materials based on ZrO_2. *Refractories and Industrial Ceramics* **1998,** *39*, 189-197.

2. Boschetto, A.; Bellusci, M.; La Barbera, A.; Padella, F.; Veniali, F., Kinematic observations and energy modeling of a Zoz Simoloyer high-energy ball milling device. *The International Journal of Advanced Manufacturing Technology* **2013,** *69* (9-12), 2423-2435.
